# Fatal Outcome in Bacteremia is Characterized by High Plasma Cell Free DNA Concentration and Apoptotic DNA Fragmentation: A Prospective Cohort Study

**DOI:** 10.1371/journal.pone.0021700

**Published:** 2011-07-01

**Authors:** Reetta Huttunen, Taru Kuparinen, Juulia Jylhävä, Janne Aittoniemi, Risto Vuento, Heini Huhtala, Janne Laine, Jaana Syrjänen, Mikko Hurme

**Affiliations:** 1 Department of Internal Medicine, Tampere University Hospital, Tampere, Finland; 2 University of Tampere Medical School, Tampere, Finland; 3 Department of Microbiology and Immunology, University of Tampere Medical School, Tampere, Finland; 4 Centre for Laboratory Medicine, Pirkanmaa Hospital District, Tampere, Finland; 5 School of Health Sciences, University of Tampere, Tampere, Finland; Institut de Pharmacologie et de Biologie Structurale, France

## Abstract

**Introduction:**

Recent studies have shown that apoptosis plays a critical role in the pathogenesis of sepsis. High plasma cell free DNA (cf-DNA) concentrations have been shown to be associated with sepsis outcome. The origin of cf-DNA is unclear.

**Methods:**

Total plasma cf-DNA was quantified directly in plasma and the amplifiable cf-DNA assessed using quantitative PCR in 132 patients with bacteremia caused by *Staphylococcus aureus*, *Streptococcus pneumoniae*, ß-hemolytic streptococcae or *Escherichia coli*. The quality of cf-DNA was analyzed with a DNA Chip assay performed on 8 survivors and 8 nonsurvivors. Values were measured on days 1–4 after positive blood culture, on day 5–17 and on recovery.

**Results:**

The maximum cf-DNA values on days 1–4 (n = 132) were markedly higher in nonsurvivors compared to survivors (2.03 vs 1.26 ug/ml, p<0.001) and the AUCROC in the prediction of case fatality was 0.81 (95% CI 0.69–0.94). cf-DNA at a cut-off level of 1.52 ug/ml showed 83% sensitivity and 79% specificity for fatal disease. High cf-DNA (>1.52 ug/ml) remained an independent risk factor for case fatality in a logistic regression model. Qualitative analysis of cf-DNA showed that cf-DNA displayed a predominating low-molecular-weight cf-DNA band (150–200 bp) in nonsurvivors, corresponding to the size of the apoptotic nucleosomal DNA. cf-DNA concentration showed a significant positive correlation with visually graded apoptotic band intensity (R = 0.822, p<0.001).

**Conclusions:**

Plasma cf-DNA concentration proved to be a specific independent prognostic biomarker in bacteremia. cf-DNA displayed a predominating low-molecular-weight cf-DNA band in nonsurvivors corresponding to the size of apoptotic nucleosomal DNA.

## Introduction

Bacteremia and sepsis are major causes of death worldwide. Despite intensive research, there are significant gaps in our knowledge regarding the basic pathophysiological mechanisms associated with fatal outcome. This hampers attempts to develop novel therapeutic interventions for this condition.

Several studies indicate that most deaths from bacteremia and sepsis are in fact the result of a substantially impaired immune response due to the extensive death of immune system cells [1]. Studies during recent years have shown that immunosupression plays a pivotal role in severe sepsis [2], [3]. The term septic immunoparalysis has been introduced [1], [2]. In clinical practice, patients with severe sepsis evince an inability to overcome initial infections and are prone to secondary hospital-acquired infections, i.e. fungal, viral and bacterial infections. Lymphocyte apoptosis, T cell anergy, decreased antigen presentation and decreased HLA-DR expression are features associated with the condition [1]. Apoptosis is an active suicidal cellular response involved in the homeostasis of cell removal under physiologic and pathologic conditions [4], [5]. It has even been debated whether immunosupression and lymphocyte apoptosis constitute rather a primary than a secondary phenomenon during severe sepsis [3]. Monitoring apoptosis on a routine basis in septic patients has been found challenging and indirect markers of apoptosis (e.g. sFas) have proved to be of limited value [4].

Circulating cell-free DNA (cf-DNA) has recently received growing attention and has been studied in various acute and chronic disorders. High cf-DNA levels have been reported in cancer [6], rheumatoid arthritis [7], stroke [8], and sepsis [9], [10] and has also been proposed as a prognostic marker in these conditions. One recent study showed increased cf-DNA concentrations in agetarians [11]. Studies in sepsis patients indicate that plasma DNA and nucleosome levels are elevated in sepsis patients with poor outcome [5], [9]. cf-DNA has been shown to predict infection [12] and in-hospital mortality in critically ill patients [10], [13]. In healthy individuals, the concentration of circulating DNA is low, as dead cells are efficiently removed from the circulation by phagocytes. Circulating DNA has a short half-life and is removed mainly by the liver. Excessive accumulation of DNA in the plasma may result from the release of DNA caused by massive cell death, inefficient removal of dying cells or a combination of both [5].

Although plasma cf-DNA in bacteremia and sepsis has been thought to be a product of accelerated cell death, the precise mechanism in septic patients remains elusive. We measured cf-DNA levels directly in plasma, quantified both total and amplifiable cf-DNA, and finally carried out a qualitative analysis of cf-DNA with a high-sensitivity lab-on-a-chip DNA assay. The results show that plasma cell free DNA (cf-DNA) is elevated in bacteremic patients with poor outcome. We demonstrate that nonsurvivors express markedly increased apoptotic DNA fragmentation. The findings presented provide novel insights into the basic pathophysiological mechanisms of a severe bacteremic infection.

## Materials and Methods

The study material comprised 132 adult patients with bacteremia admitted to Tampere University Hospital, Tampere, Finland, from June 1999 to February 2004 (Table 1). Patient recruitment, clinical data collection and sample collection were prospective. Samples for cf-DNA were analyzed after hospitalization.

**Table 1:** Characteristics of the study population.

Characteristic	
Causative organism	
S. aureus	32 (24%)
Str. pneumoniae	37 (28%)
B- hemolytic streptococcus	22 (17%)
E. coli	41 (31%)
Demographic features and underlying diseases	
Age, median (range)	62 (16–93 years)
Gender (female/male)	62/70
Cancer (solid or hematological)	23 (17%)
At least one chronic disease	107 (81%)
Alcohol abuse	21 (16%)
Diabetes mellitus (type 1 or 2)	33 (25%)
BMI (kg/m^2^), median (range)^a^	26 (15–39)
Bacteremia focus	
Skin	33 (25%)
Urinary	29 (22%)
Lung	33 (25%)
Osteomyelitis/spondylitis	13 (10%)
Other or unknown focus	41 (31%)

a BMI data available on 101 patients.

In our hospital blood cultures are routinely taken in cases with symptoms or signs of systemic infection (fever or hypothermia, tachycardia or tachypnea combined with leucocytosis or leucopenia and/or elevated C-reactive protein (CRP)). The BACTEC 9240 (BD Diagnostic Systems, Sparks, MD, USA) blood culture system was used with standard media. Patients were identified according to microbiological blood culture finding, and only those with bacteremia caused by *S. aureus, Str. pneumoniae*, ß-hemolytic streptococcus or *E. coli*, the most common causative organisms in community-acquired bacteremia, were included in the study, other microbes being excluded beforehand. Blood culture-negative patients with or without sepsis syndrome and those not consenting were not included. All patients included in the study had verified infection. Only patients at least 16 years of age were enrolled. The clinicians (J.S. or J.L.) were informed by the clinical microbiologist (R.V.) of a positive blood culture from Mondays to Thursdays and the patients were enrolled in the study whenever possible to adjust to the daily schedule. We were able to recruit zero to two patients per week during the study period. Since the clinicians had no knowledge of details regarding the patients or their disease severity prior to recruitment, selection was based solely on the blood culture finding. Upon notification by the clinical microbiologist the clinicians (J.L. and J.S.) asked patients to participate and interviewed and examined those consenting. Information was gathered from hospital records at the time of a hospital visit and hospital records were also reviewed subsequent to hospitalization (R.H.). Altogether 149 out of 152 patients agreed to participate. Samples for cf-DNA determinations during 1–4 days after positive blood culture were available in 132 patients, and these were recruited as the final study population. The study was approved by the Ethics Committee of Tampere University Hospital and written informed consent was obtained from patients or first-degree relatives. All subjects were treated with an empiric antibiotic regimen, and when necessary antimicrobial treatment was changed according to culture results. In all patients the causative organism proved susceptible to the first empiric antibiotic treatment selected on admission.

### Underlying conditions and chronic diseases

Chronic diseases and sources of bacteremia were registered. Alcohol abuse was defined as consumption of 300 g absolute alcohol per week or a known social or medical problem due to alcohol use. Patients were defined as current smokers and nonsmokers, i.e. those who had never smoked or had stopped smoking. Calculation of body mass index (BMI, kg/m^2^) was based on weight and height as reported by the patient on admission. Patients were defined as obese if their BMI was ≥30 kg/m^2^. McCabe classification [14] was used to determine the severity of underlying diseases.

### Clinical data and laboratory tests

Clinical data and laboratory findings were registered on admission and during 6 consecutive days. Alterations in mental status were evaluated on the Glasgow Coma Scale (GCS) and possible mechanical ventilation and the need for intensive care unit (ICU) treatment were recorded. Mean arterial pressure (MAP) ((systolic+2 x diastolic blood pressure)/3) and SOFA score (sequential organ failure assessment) [15] were calculated. The maximum SOFA score (days 0–6) for every patient was used in analysis. Disease severity was assessed by SOFA score, severe disease being defined as a score ≥4. Laboratory tests included plasma C-reactive protein (CRP, mg/l), blood platelets (×10^9^/l), plasma bilirubin (µmol/l), plasma creatinine level (µmol/l) and blood leucocyte count (×10^9^/l). We have recently published a study on the prognostic value of high kynurenine (kyn) to tryptophan (trp) concentration ratio detected on days 1 to 4, reflecting indoleamine 2,3 dioxygenase (IDO) activity [16]. Kyn and trp values had been examined by high-performance liquid chromatography (HPLC) [16]. The optimal cut-off for maximum IDO activity in predicting fatal disease had been evaluated using the receiver operating characteristic curve (ROC)[16]. The case fatality rate was studied within 14 and 30 days after a positive blood culture (d-14 and d-30 case fatality).

Samples were available for altogether 132 patients on days 1–4 after the positive blood culture. Multiple samplings in the same patient were always performed on separate days. Samples for cf-DNA determinations were available on day 1–2 (1–2 days after the blood culture was taken): 34 patients, on day 3∶ 81 patients and on day 4∶ 104 patients. In addition, 121 patients gave a sample on day 5–17 (5–17 days after blood culture) and 89 gave a sample on recovery (>25 days after positive blood culture). The maximum values for cf-DNA in every patient measured during 1–4 days after positive blood culture were determined. Since patient recruitment was based on blood culture, which only became positive the following day, no samples for cf-DNA were available on day 0 (blood culture day).

### Quantification of total plasma cf-DNA

The amount of total cf-DNA was determined directly in plasma without any purification step, using the Quant-iT™ high-sensitivity DNA assay kit and a Qubit® fluorometer (Invitrogen, Carlsbad, CA, USA) following the manufacturer's instructions. The assessed intra-day variation coefficients at the mean cf-DNA levels of 0.734 µg/ml, 1.377 µg/ml and 4.954 µg/ml were 1.8%, 4.3% and 1.7%, respectively. The corresponding inter-day variation coefficients were 3.8%, 5.0% and 3.2%.

### Extraction and qualitative analysis of cf-DNA

Qualitative analysis of cf-DNA was performed in randomly selected (n = 16) cases (n = 8 survivors and n = 8 non-survivors). Plasma cf-DNA was extracted using the NucleoSpin® Plasma XS Kit (MACHEREY-NAGEL GmbH & Co., Düren, Germany), designed for isolation of low-molecular-weight (50–1000 bp) cf-DNA. Cf-DNA isolation was performed according to the manufacturer's instructions following the high-sensitivity protocol. Extracted cf-DNA samples were stored at -70°C until further analyses.

Extracted cf-DNA samples were analyzed with the High Sensitivity DNA assay kit and an Agilent 2100 Bioanalyzer equipped with Expert 2100 software according to the manufacturer's instructions (Agilent Technologies Inc., Santa Clara, CA). The Agilent 2100 Bioanalyzer is an instrument which uses a lab-on-a-chip technology to perform gel electrophoresis; nucleic acids are separated analogously to capillary electrophoresis and normalized to a ladder and two DNA markers, whereafter the software automatically calculates the size of each band. For each sample, the appearance and intensity of low-molecular-weight cf-DNA was estimated visually and graded as follows: 1 = no visible cf-DNA or weak intensity, 2 = intermediate intensity, 3 = strong intensity. The researcher responsible for analyzing and grading the cf-DNA samples was blinded to the outcome of the patient.

### sFas and Fas ligand measurements

Human soluble Fas (sFas) and human Fas ligand concentrations in plasma were determined using commercial quantitative sandwich enzyme immunoassays (Quantikine®, R&D Systems Inc., Minneapolis, MN, USA.

### Statistical analysis

An SPSS package (version 7.5 and version 10) was used for statistical analyses and a two-sided p-value <0.05 was taken as cut-off for statistical significance. Categorical data were analyzed by *X^2^* test or Fisheŕs exact test when appropriate, nonparametric data by Mann-Whitney U-test or Kruskal-Wallis test. A logistic regression model was used to study the independent effect of high cf-DNA concentration on mortality models adjusted for potential confounders. Odds ratios (ORs) were expressed with their 95% confidence intervals (CI). The survival curve was calculated using the Kaplan-Meier method and survival differences between groups were compared using the log rank test. The accuracy of maximum cf-DNA in predicting case fatality was evaluated using ROC curves [17]. In this method, a test which is perfect has 100% sensitivity and no false-positives (1-specificity = 0) and will have an area under the curve (AUC) of 1.0, whereas a test of no diagnostic value would have an AUC of 0.5. Youden index with the highest sum of sensitivity and specificity (sensitivity + (1-specificity)) was used to select optimal cut off for analysis. Spearman's Rank correlation test was used to test the direction and strength of the relationship between two variables. The total amount of cf-DNA (Quant-iT™ assay) was correlated with the graded intensity of the low-molecular-weight cf-DNA, sFas, Fas ligand and kyn/trp ratio using Spearman's ρ.

## Results

Baseline characteristics of bacteremia patients are shown in Table 1. The median plasma cf-DNA value in the acute phase (maximum value 1 to 4 days after blood culture) was 1.29 µg/ml (quartiles 1.13–1.69 µg/ml) and 1.19 µg/ml (quartiles 1.03–1.43) on days 5–17 after blood culture. The median value >25 days after blood culture was 0.88 µg/ml (quartiles 0.78–0.98 µg/ml). cf-DNA values in patients with bacteremia stratified by demographics, underlying conditions and causative organism are shown in Table 2. Of chronic conditions, alcohol abusers had higher cf-DNA values compared to patients without the history of alcohol abuse (Table 2).

**Table 2:** Plasma cell free DNA (cf-DNA) values in patients with bacteremia stratified by various demographic features, underlying conditions and causative organism.

Character	Maximum cf-DNA (ug/ml) 1 to 4 days after blood culture		p-value
	Stratification by underlying factors		
	Factor present, median (quartiles)	Factor absent, median (quartiles)	
Age over 60 years	1.28 (1.11–1.72)	1.31 (1.15–1.65)	0.796
Male	1.35 (1.19–1.87)	1.24 (1.09–1.41)	0.031
Obesity (BMI≥30 kg/m2)^a^	1.35 (1.21–2.00)	1.27 (1.11–1.61)	0.252
Current smoking^b^	1.41 (1.18–2.00)	1.27 (1.11–1.46)	0.096
Alcohol abuse	1.61 (1.31–2.69)	1.26 (1.10–1.53)	0.001
Diabetes mellitus type 1 or 2	1.33 (1.05–1.80)	1.28 (1.15–1.66)	0.787
Cancer	1.29 (1.07–1.66)	1.28 (1.14–1.71)	0.760
McCabe class II or III^c^	1.36 (1.11–1.76)	1.38 (1.13–1.68)	0.656
**Causative organism**			
*S. aureus*	1.40 (1.28–1.96)		
*Str. pneumoniae*	1.28 (1.14–1.71)		0.011^d^
B-hemolytic streptococcus	1.19 (1.02–1.62)		
*E. coli*	1.24 (1.09–1.45)		

a BMI data available on 101 patients,

b smoking data available on 120 patients,

c ultimately or rapidly fatal disease,

d value indicates the difference in cf-DNA values between groups of patients stratified by different causative organism.

### cf-DNA and outcome of bacteremia

**Figure 1:**
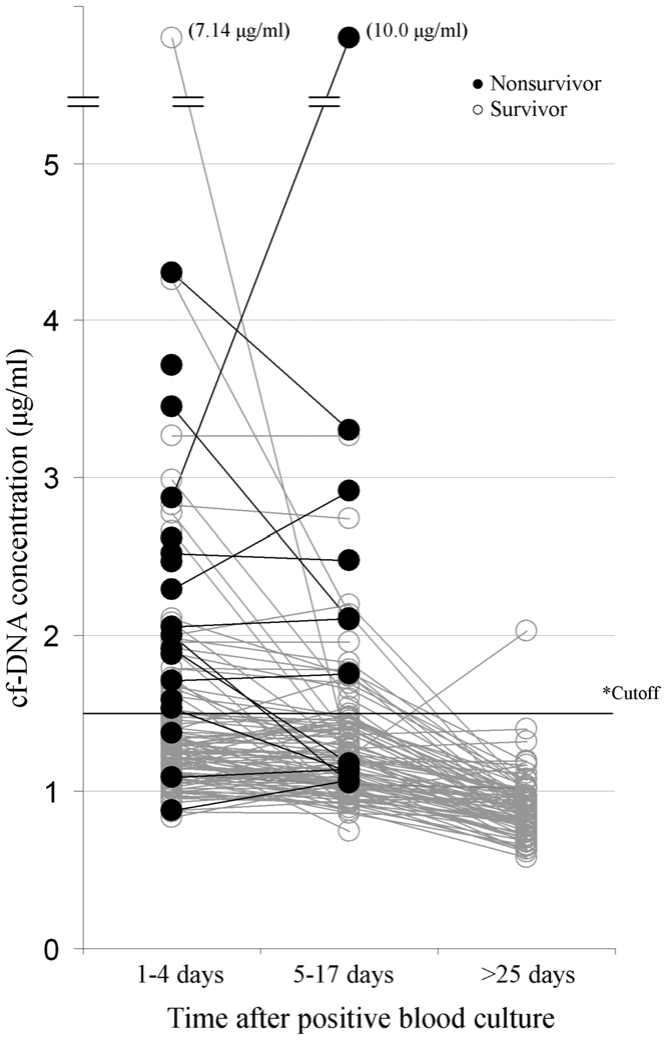
cf-DNA line plot diagram. A Line plot diagram showing cell free DNA (cf-DNA) concentration detected on days 1–4, 5 to 17 days and >25 days after blood culture in nonsurvivors (black plots) and in survivors (open plots).

Median cf-DNA values were significantly higher in nonsurvivors compared to survivors on days 3 (1.97 vs 1.20 ug/ml, p<0.001) and on day 4 (1.91 vs 1.25 ug/ml, p<0.001) after the initial diagnosis (blood culture day) (Table 3). Maximum cf-DNA values on days 1 to 4 after the initial diagnosis (blood culture day) were significantly higher in nonsurvivors compared to survivors (median values 2.03 and 1.26, p<0.001) (Figure 1).

**Table 3:** Plasma cell free DNA concentration during days 1 to 4 after blood culture in relation to bacteremia outcome.

Days after blood culture	Plasma cell free DNA (ug/ml), median (quartiles)		p-value
	Nonsurvivors n = 18	Survivors n = 118	
**Day 1–2**	1.36 (1.31–1.81)	1.27 (1.10–1.44)	0.166
**Day 3**	1.97 (1.59–2.38)	1.20 (1.06–1.37)	<0.001
**Day 4**	1.91 (1.49–2.70)	1.25 (1.11–1.38)	<0.001
**Maximum value (days 1 to 4)**	2.03 (1.57–2.68)	1.26 (1.11–1.47)	<0.001

a cf-DNA values available on 34 patients on day 1–2, 81 patients on day 3, 104 patients on day 4 and 132 patients on day 1 to 4 (maximum value).

**Figure 2:**
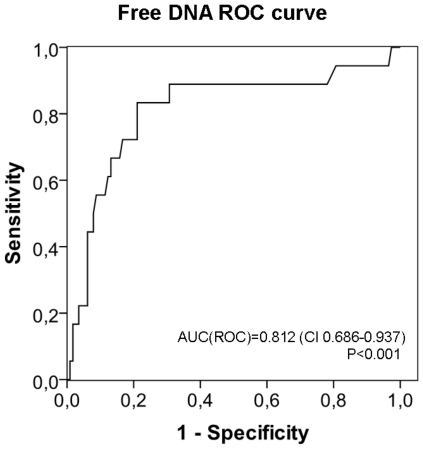
cf-DNA ROC curve. Receiver operating characteristic (ROC) curve for maximal plasma cell free DNA (cf-DNA) concentration detected on days 1–4 after positive blood culture in relation to case fatality in bacteremia patients.

**Figure 3:**
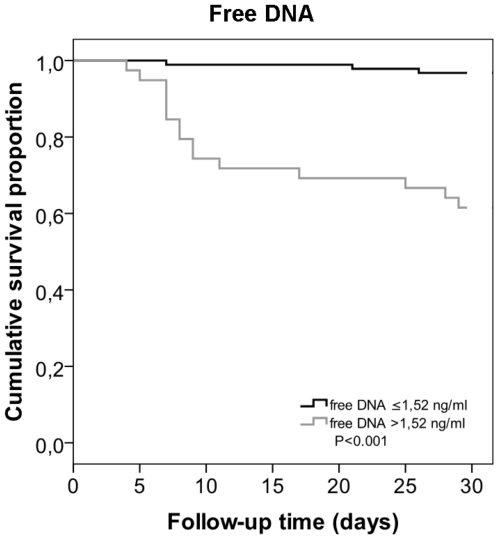
cf-DNA and survival curve. Cumulative 30-d survival in bacteremia patients with maximum plasma cell free DNA (cf-DNA) >1.52 ug/ml compared to those with cf-DNA ≤1.52 ug/ml. The cut off value of 1.52 ug/ml was chosen using ROC curve. The survival curve was calculated using the Kaplan-Meier method, and survival differences between groups were compared by log-rank test.

The optimal cut-off value for the maximum cf-DNA values on days 1–4 in predicting fatal disease was estimated using ROC curve, illustrated in Figure 2. The cf-DNA value at a cut-off level of 1.52 ug/ml showed a sensitivity of 83% and a specificity of 79% in detecting fatal disease, and this cut-off point was used to classify patients into those with high or low cf-DNA value. High cf-DNA values were associated with several endpoints indicative of severe disease (Table 4). Figure 3 shows the cumulative 30-d survival in bacteremia patients with maximum plasma cf-DNA level (1–4 days after blood culture) >1.52 ug/ml compared to those with ≤1.52 ug/ml.

**Table 4:** Clinical disease severity of patients stratified by maximum plasma cell free DNA (cf-DNA) value (1 to 4 days after blood culture).

Clinical disease severity	High cf-DNA (>1.52 ug/ml) N = 39	Low cf-DNA (≤1.52 ug/ml) n = 93	OR (95% CI)	p-value
**Grouping variables**				
Died (d-30 case fatality)	15 (39%)	3 (3%)	18.8 (5.0–70.1)	<0.001
Died (d-14 case fatality)	11 (28%)	1 (1%)	36.1 (4.5–292.3)	<0.001
Hypotensive	27 (69%)	25 (27%)	6.1 (2.7–13.9)	<0.001
Needed ICU stay^a^	24 (62%)	18 (19%)	6.7 (2.9–15.2)	<0.001
Needed vasopressives	21 (54%)	5 (5%)	20.5 (6.8–61.6)	<0.001
Lowered Glasgow coma scale (<15)	26 (67%)	27 (29%)	4.9 (2.2–10.9)	<0.001
Needed mechanical ventilation	15 (39%)	5 (5%)	11.0 (3.6–33.3)	<0.001
Highest SOFA score≥4^b^	30 (77%)	25 (27%)	9.1 (3.8–21.7)	<0.001
**Continuous variables**				
Minimum MAP^c^ (mmHg), median (quartiles)	62 (52–73)	77 (68–90)		<0.001
Maximum SOFA score, median (quartiles)	9 (4–13)	2 (0–4)		<0.001
Maximum bilirubin level, median (quartiles)	33 (17–81)	16 (12–25)		<0.001
Maximum creatinine level, median (quartiles)	159 (95–213)	96 (73–167)		0.005
Median neutrophil count (×10^9^/l) (quartiles) (n = 112)	10.3 (7.4–13.3)	6.8 (3.9–9.5)		0.002
Maximum C-reactive protein (mg/l)	279 (204–372)	213 (150–329)		0.025
Minimum platelet count (×10^9^/l), median (quartiles)	86 (33–165)	181 (116–241)		<0.001

a intensive care unit,

b sequential organ failure assessment,

c mean arterial pressure, $continuous variable (OR and CI cannot be applied).

**Figure 4:**
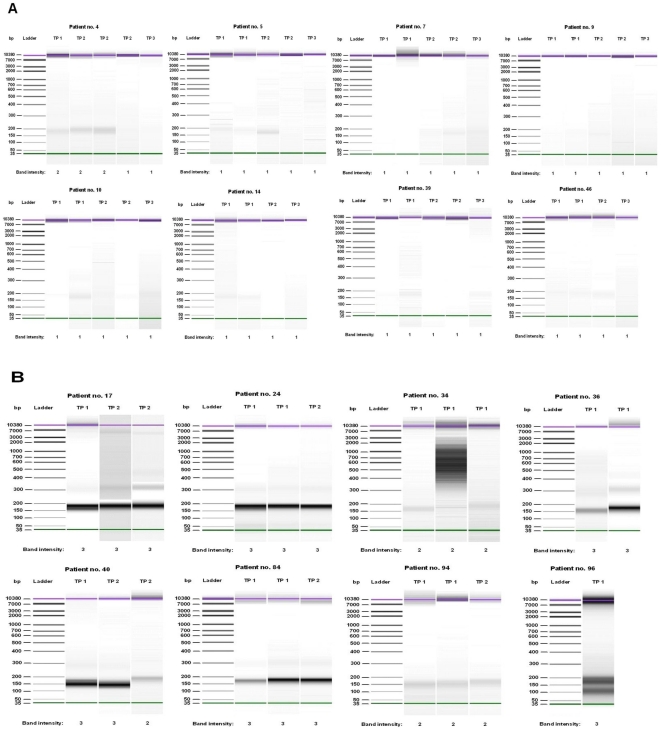
Qualitive cf-DNA analysis. Qualitative analysis of plasma cf-DNA in 8 bacteremia survivors (panel A) and 8 non-survivors (panel B) after NucleoSpin® Plasma XS kit extraction. Analyses were performed with Agilent's High Sensitivity Lab-on-a-chip DNA assay. Green lines indicate the low-weight (35 bp) DNA marker and purple lines the high- weight (10 380 bp) DNA marker. TP 1 = time point 1 (1 to 4 days after blood culture), TP 2 =  time point 2 (5 to 17 days after blood culture), TP 3 = time point 3 (recovery, >25 days after blood culture). The intensity of the low-molecular-weight cf-DNA band was graded as follows: 1 = no visible cf-DNA or weak intensity, 2 = intermediate intensity, 3 = strong intensity. Abbreviations; bp =  base pairs, TP = time point.

Qualitative analysis of cf-DNA revealed that cf-DNA displays a predominating low-molecular- weight cf-DNA band (150–200 bp) in nonsurvivors corresponding to the size of apoptotic nucleosomal DNA (Figure 4). Spearman′s correlation test showed a significant positive correlation between the acute phase (1 to 4 days after blood culture) cf-DNA level and visual grading of apoptotic band intensity (R = 0.822, p<0.001)(Table 5). A weak, albeit significant, positive correlation between cf-DNA and sFas (ug/ml) was documented (Table 5).

**Table 5:** Spearman′s test correlation between cf-DNA level and C-reactive protein (CRP), soluble Fas (sFas), Fas ligand (FasL), kynurenine to tryptophan ratio (kyn/trp) and visually graded band intensity in gel electrophoresis.

	cf-DNA	
Test	Correlation coefficient	p-value
C-reactive protein (mg/l)	0.217	0.012
sFas (ug/ml) (n = 132)	0.383	<0.001
Fas ligand (ug/ml) (n = 132)	−0.183	0.036
Kyn/trp ratio (µmol/mmol)(n = 132)	0.400	<0.001
Visually graded apoptotic 200 bp band intensity (I–IV) (n = 16)	0.822	<0.001

Acute phase values (during days 1 to 4 after blood culture) were studied.

The independent effect of high (>1.52 ug/ml) maximum cf-DNA value on case fatality was studied in a logistic regression model adjusted for potential confounders. The following grouping variables have previously been shown to be associated with case fatality in a univariate model in this material: obesity, smoking, alcohol abuse and high SOFA score (≥4)[16], [18]. High cf-DNA detected on days 1–4 after blood culture retained its significance in the logistic regression model in all combinations. High maximun cf-DNA value was studied together with one possible confounder at a time in the model, as there were only 18 patients who died. cf-DNA predicted outcome independently of alcohol abuse, gender and causative organism (data not shown). Obesity and high SOFA score (≥4) also remained independent factors associated with case fatality when studied together with high cf-DNA.

## Discussion

The present results show that the plasma cell free DNA concentration is significantly higher in bacteremia nonsurvivors compared to survivors. Qualitative analysis of cf-DNA revealed that cf-DNA displays a predominating low-molecular-weight cf-DNA band (150–200 bp) in nonsurvivors corresponding to the size of apoptotic nucleosomal DNA.

The present findings are in accord with those of previous studies showing increased levels of plasma cf-DNA in septic patients. The present study provides novel evidence regarding the origin of cf-DNA from apoptotic cells. This finding is line with the suggestion that severe sepsis is characterized by apoptotic cell death and that this phenomenon plays a pivotal role in fatal cases [1]. Apoptosis is a form of cell death characterized by cytoplasmic condensation, compaction of nuclear chromatin and nuclear fragmentation, resulting in a characteristic pattern of DNA laddering in agarose gel electrophoresis. Recent studies indicate upregulation of the pro-apoptotic genes BID and FAS in septic shock patients [19]. Animal studies suggest that blocking apoptosis may constitute an intriguing opportunity to improve outcomes in sepsis [1]. Caspase inhibition is a novel sepsis therapy in preclinical development, based on blocking apoptosis [1].

Circulating DNA in plasma is protein-bound (nucleosomal) DNA. Quantification of circulating DNA can be performed by real-time quantitative PCR or immunological methods such as ELISA [11]. Circulating DNA is known to be highly fragmented and low in concentration, which creates difficulties in its purification. In the present study, the amount of total cf-DNA was determined directly in plasma without any purification step using a high-sensitivity DNA assay kit and a fluorometric method. As purification was not needed for quantification, this approach constitutes a rapid and cheap means of detecting free DNA in plasma. In oncology, cf-DNA has been shown to be useful in staging, identifying disease progression and response to therapy in patients with cancer [8]. CRP has been shown to be of limited prognostic value in the present cohort [16]. Thus, cf-DNA measurement may be useful in clinical use and stratification of patients with distinct bacteremia outcomes.

Previous studies have studied cf-DNA concentrations in sepsis expecially in ICU settings [9], [10]. Saukkonen and associates showed that cf-DNA was an independent predictor of ICU but not in-hospital mortality in severe sepsis and septic shock [9]. Several studies have shown cf-DNA to predict outcome in all-cause ICU patients [10], [13]. One study showed that cf-DNA predicted the presence of infection in febrile patients and that cf-DNA also predicted outcome [12]. Rhodes and associates showed that high plasma cf-DNA predicted the development of sepsis or septic shock in critically ill patients [10]. Studies in cancer patients indicate that circulating DNA originates from apoptotic or necrotic cells [6], reflecting the extent of cellular damage. Supporting such a conception, hypothesis, cf-DNA has frequently been observed with a nucleosomal (150–200 base pairs in length) or a ladder-like appearance [6], [20]. To the best of our knowledge, the present study shows for the first time that cf-DNA expresses apoptotic fragmentation in bacteremia nonsurvivors. The mechanisms underlying apoptotic cell death in sepsis remain elusive. Imbalance between oxygen delivery and consumption resulting in anaerobic glycolysis and lactate production are central features in severe septic infection. This may lead to oxygen deprivation, endothelial cell damage and subsequent apoptotic cell death. Interestingly, cf-DNA levels were higher in males than in females in the acute phase. Male sex did not however predict case fatality and cf-DNA remained a significant factor associated with case fatality after adjusting for patient gender. We demonstrate a significant difference in cf-DNA values between groups of patients stratified by different causative agents. Bacteremias caused by different organisms have been shown to have different case fatality rates and evidence suggests that the host response may differ depending on the type of microbial pathogen [21].

We showed a weak, albeit significant, correlation with the indirect marker of apoptosis, sFas and with kynurenine to tryptophan ratio reflecting IDO activity. A correlation with sFas and cf-DNA is in line with the fact that the progression of apoptosis is regulated by intracellular signaling pathways after binding of a death-specific receptor (fas) to its ligand (fasl) [4]. We recently published a study on the prognostic value of high IDO concentration ratio and showed an association between high IDO and case fatality in bacteremia [16]. IDO is an enzyme which degrades the essential amino acid tryptophan to kynurenine, with subsequent suppression of T-cells [22]. IDO has been shown to have a central role in the regulation of blood pressure in sepsis [23]. It remains to be established whether IDO contributes to immune system paralysis in bacteremia and sepsis.

Some limitations have to be considered here. Due to the study design, the potential causal role of cf-DNA in fatal cases could not be adressed. Previous studies indicate that precisely lymphocytes are prone to apoptotic cell death in severe sepsis. Apoptosis has been shown to take place both in the gastrointestinal tract epithelium and in circulating blood lymphocytes. Further work is needed to investigate the intracellular sources of plasma DNA. No control group was used in the present study, which constitutes a limitation. Controls from critically ill patients with no bacteremia or sepsis would have been important to indicate cf-DNA values in these patients. Previous studies suggest that infection *per se* is associated with higher cell-free plasma DNA concentrations compared to non-infectious conditions [12], [13]. However, the present findings consistently showed median cf-DNA levels to be higher in acute illness than in the convalescence phase. We studied most common bacterial pathogens encountered in the case of community-acquired bacteremia. The present findings can be generalized to bacteremias caused by *S. aureus*, *E. coli*, ß-hemolytic streptococcus and *Str. Pneumoniae*. Thus, the significance of cf-DNA as a predictor of case fatality should be studied also in bacteremias caused by other culprit organisms in the future.

In conclusion, it was established here that plasma cf-DNA concentrations are significantly increased in bacteremia patients with poor outcome. Nonsurvivors express apoptotic DNA fragmentation bands. These findings support the conception that apoptosis plays a pivotal role in severe bacteremic infection. The development of therapeutic interventions targeted to apoptosis pathways may be a key element leading to improved patient outcomes. cf-DNA can be used as a non-invasive, rapid, sensitive and accurate marker of severe bacteremia.
